# The spatial variability of NDVI within a wheat field: Information content and implications for yield and grain protein monitoring

**DOI:** 10.1371/journal.pone.0265243

**Published:** 2022-03-22

**Authors:** Paul C. Stoy, Anam M. Khan, Aaron Wipf, Nick Silverman, Scott L. Powell

**Affiliations:** 1 Department of Biological Systems Engineering, University of Wisconsin–Madison, Madison, WI, United States of America; 2 Nelson Institute for Environmental Studies, University of Wisconsin–Madison, Madison, WI, United States of America; 3 Department of Land Resources and Environmental Sciences, Montana State University, Bozeman, MT, United States of America; 4 Adaptive Hydrology LLC, Missoula, MT, United States of America; Institute of Genetics and Developmental Biology Chinese Academy of Sciences, CHINA

## Abstract

Wheat is a staple crop that is critical for feeding a hungry and growing planet, but its nutritive value has declined as global temperatures have warmed. The price offered to producers depends not only on yield but also grain protein content (GPC), which are often negatively related at the field scale but can positively covary depending in part on management strategies, emphasizing the need to understand their variability within individual fields. We measured yield and GPC in a winter wheat field in Sun River, Montana, USA, and tested the ability of normalized difference vegetation index (NDVI) measurements from an unoccupied aerial vehicle (UAV) on spatial scales of ~10 cm and from Landsat on spatial scales of 30 m to predict them. Landsat observations were poorly related to yield and GPC measurements. A multiple linear model using information from four (three) UAV flyovers was selected as the most parsimonious and predicted 26% (40%) of the variability in wheat yield (GPC). We sought to understand the optimal spatial scale for interpreting UAV observations given that the ~ 10 cm pixels yielded more than 12 million measurements at far finer resolution than the 12 m scale of the harvester. The variance in NDVI observations was “averaged out” at larger pixel sizes but only ~ 20% of the total variance was averaged out at the spatial scale of the harvester on some measurement dates. Spatial averaging to the scale of the harvester also made little difference in the total information content of NDVI fit using Beta distributions as quantified using the Kullback-Leibler divergence. Radially-averaged power spectra of UAV-measured NDVI revealed relatively steep power-law relationships with exponentially less variance at finer spatial scales. Results suggest that larger pixels can reasonably capture the information content of within-field NDVI, but the 30 m Landsat scale is too coarse to describe some of the key features of the field, which are consistent with topography, historic management practices, and edaphic variability. Future research should seek to determine an ‘optimum’ spatial scale for NDVI observations that minimizes effort (and therefore cost) while maintaining the ability of producers to make management decisions that positively impact wheat yield and GPC.

## Introduction

Crop yields are often quite variable within individual fields due to differences in soil fertility, topography, weediness, and management efforts, but also for reasons that are not entirely clear [1]. Canopy spectral reflectance indices like the normalized difference vegetation index (NDVI) are useful for estimating crop yield within individual fields [2–4] because the absorption and reflectance of red and near-infrared wavelengths is a good proxy for leaf area, which in turn is a good proxy for growth [5] and yield [6]. Following this notion, the yields of many different crops have been estimated using NDVI and related vegetation indices using aerial and satellite-based platforms [7–9].

Other crop attributes also determine price, like grain protein content (GPC) for the case of wheat (*Triticum aestivum* L.) [10,11]. Understanding GPC is critical not only for agricultural management [12] but also for the global food system as it is predicted to decrease in a changing climate [13]. Wheat yield and GPC are often inversely related within a field [14–17] because water stress during grain filling increases GPC but decreases yield [18]. Despite this, yield and GPC can be positively related depending on edaphic properties and management interventions [16,19], with great advantage to producers. Field-scale management can therefore be improved by understanding relationships between NDVI, yield, and GPC.

The spatial variability of GPC has been successfully estimated from NDVI and other vegetation indices using different remote sensing platforms [20–26] and across time [27,28], especially during later stages of crop development, namely anthesis [18,29,30]. Wheat yield is often more strongly related to vegetation indices that are integrated across the growing season to capture the full period of canopy development and thereby crop carbon uptake [31–33]. As with all remote sensing products, there is a tradeoff between frequent measurements and spatial resolution that needs to be understood when designing observation systems. Satellite platforms offer frequent observations at scales of tens of meters to kilometers, which may be insufficient to capture within-field spatial variability. Unoccupied aerial system technologies and portable spectroradiometers [34] can collect observations at spatial scales on the order of centimeters or less [35] but usually make measurements rather infrequently, depending on effort, which adds cost. Wheat yield and GPC can even be estimated using consumer-grade cameras [36] that can be mounted as ‘phenocams’ to take repeat measurements at frequent intervals at fine spatial scales [37]. With these emerging technologies and opportunities, an important question remains: in a data-rich world, what observations are necessary for a concise description of within-field variability of wheat yield and GPC? We argue that the answer lies, in part, in understanding the patterns of spatial variability of NDVI, yield, and GPC within wheat fields.

Here, we investigate the relationships between wheat yield and GPC measured by a harvester, NDVI observations from an unoccupied aerial vehicle (UAV) at the scale of approximately 12.5 cm, and NDVI observations at 30 m from Landsat. We ask if the spatial scale of Landsat is sufficient to characterize field-scale variability in wheat yield and GPC and, hypothesizing that it is not, seek to understand which UAV-based observations create the best fit with both yield and GPC observations. We then quantify the consequences of spatial averaging on NDVI statistics and information loss to quantify the compromises that one makes by observing at coarser spatial resolution. We discuss our findings in the context of field-scale management and ways to efficiently use spatial data to improve wheat yield and GPC.

## Materials and methods

### Study site

**Fig 1:**
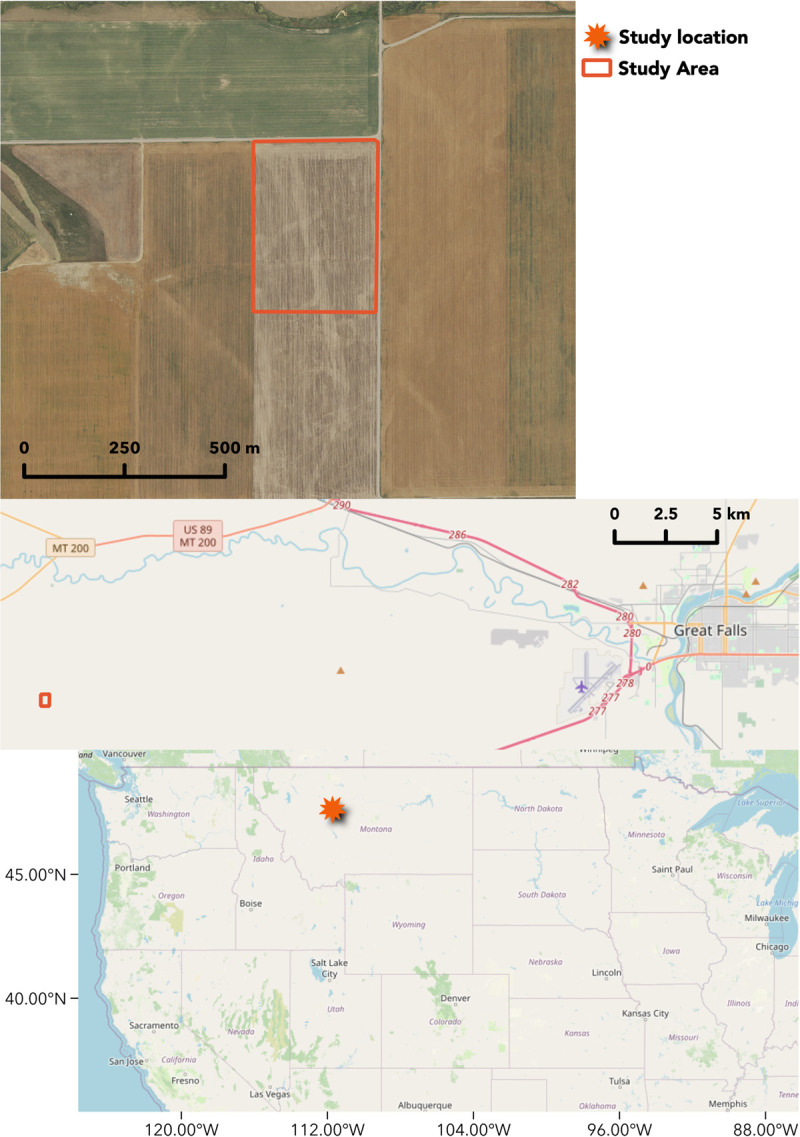
(top) A map of the study area; a winter wheat field near Sun River, Montana, USA. Aerial imagery was acquired on July 3, 2017, from the National Agricultural Imagery Program. The two base maps and data displayed in the top figures are from OpenStreetMap and OpenStreetMap Foundation (CC-BY-SA) and contributors, https://www.openstreetmap.org.

Measurements were made in an agricultural field located south of Sun River, Montana, USA (Fig 1) [38] that housed the “US-MSR” Ameriflux eddy covariance measurement tower at 47.4758 N, 111.7207 W (see S1 Fig). The mean annual temperature over the past 30 years at the Great Falls International Airport located 25 km due east of the study site is 7.0°C and the mean annual precipitation is 375 mm. The study area is 420 m in the east-west direction and 570 m in the north-south direction with rows oriented north-south. Brawl CL Plus hard red winter wheat [39] was planted in 2015 and harvested in 2016 following a year of summer fallow in 2015, winter wheat harvested in 2014, a combination of pea (*Pisum sativum*), lentil (*Lens culinaris*), and mustard (*Brassica hirta*) harvested in 2013, and summer fallow in 2012.

### NDVI acquisition and analysis

We acquired multispectral imagery on May 19, June 8, July 1, and July 20, 2016, between 900 and 1400 local standard time to minimize sun angle effects, with most flights occurring within an hour of 1000. Observations from the different dates are subsequently abbreviated NDVI_date_. We first established eight permanent ground control points using a R8-3 base station and a R8-4 multi-constellation GNSS receiver (Trimble, Sunnyvale, CA, USA) and achieved 1.5 to 1.8 cm precision at a 95% confidence interval in both the horizontal and vertical directions. Green (550 nm), red (660 nm), red edge (735 nm), and NIR (790 nm) bands were measured using a senseFly multiSPEC 4C camera mounted on an eBee drone (senseFly Ltd., Cheseaux-Lausanne, Switzerland) with integrated inertial measurement unit, global positioning system (GPS), and autopilot. The multiSPEC 4C camera contains an integrated upward-facing irradiance sensor, which was calibrated before each flight with an Airinov MultiSPEC 4C calibration target. This allowed us to convert spectral radiance to reflectance and compare NDVI among measurement dates. SenseFly eMotion 2 software was used for flight planning, execution, and preliminary processing. Othomosaics and NDVI rasters for each date were derived by post-processing with Pix4Dmapper Pro (Pix4D SA, Lausanne, Switzerland). The average ground sampling distance was 12.5 cm with an average geolocation root mean square error (RMSE) of 2.3 cm (Table 1). Observations were resampled to match the spatial scale of the image with the coarsest resolution, 13.43 cm from the July 1 image. We created a daily NDVI product for the May 19—July 20 period, NDVI_int_, by linearly interpolating NDVI observations from each pixel from each UAV flight.

**Table 1:** Average ground sampling distance (GSD, i.e. ‘pixel size’) and the root mean square error (RMSE) of the ground control point used for UAV imagery on each date.

Date (2016)	GSD (cm)	Geolocation RMSE (cm)
May 19	11.03	3.6
June 8	12.48	1.4
July 1	13.43	2.6
July 20	13.13	1.7

### Landsat

Landsat NDVI calculations were made at 30-meter resolution using data from the Landsat 7 mission and Google Earth Engine [40]. We used the maximum NDVI value for the calendar year to compare with yield data from the combine harvester.

### Data analysis

#### Unsupervised classification

We combined the four dates of UAV NDVI imagery into a single raster file for spatio-temporal classification. We used k-means unsupervised classification in Erdas Imagine (Hexagon Geospatial, Norcross, GA), with 50 initial classes. From these data, we used the Grouping Tool to create three classes from the 50 original classes using expert knowledge of the field (topography, geology, soil distribution, etc.) to logically combine classes. We then imported the three-class classified map into ArcMap (Esri, Inc., Redlands, CA), created masks for each group, and extracted the NDVI values for each of the four dates. We averaged the NDVI values for each date and class to create four-date trajectories of average NDVI.

#### Comparison of NDVI to yield data

Georeferenced (“GPS-tagged”) wheat yield and GPC measurements were made using a combine yield monitor during harvest (S2 Fig). These data were cleaned using Yield Editor (United States Department of Agriculture, Washington D.C.) to adjust for sensor lag and missing values. To match the footprint of the combine with observed NDVI values, we created 1×12 m rectangular buffers around each yield point, from which we extracted the average NDVI values from each date within the buffer polygon.

#### Statistical analysis

We used Akaike’s Information Criterion (AIC) to select amongst different linear models of yield and GPC as a function of NDVI measured on the four different dates as well as NDVI_int_. Models were selected using the *dredge* routine in the MuMIn package [41] in R [42].

#### Spatial analysis

We calculated the change in total variance of NDVI that results from averaging with increasingly large pixels to understand how variance is “averaged out” at coarser spatial scales, often called the ‘grain’ of the image, not to be confused with the grain crop. NDVI varies between 0 and 1 in the absence of water bodies and, if unimodal, can be modeled as a Beta distribution [43] as increasingly used for studies of plant cover [44]. We fit Beta distribution parameters using observations from the original images and the spatially-averaged images using maximum likelihood methods. We then calculated the change in information content that results from spatial averaging using the Kullback-Leibler divergence (D_KL_) for the Beta distribution:

$$D_{KL}=ln(\frac{B(\alpha \prime ,\beta \prime )}{B(\alpha ,\beta )})+(\alpha -\alpha \prime )\psi (\alpha )+(\beta -\beta \prime )\psi (\beta )+(\alpha \prime -\alpha +\beta \prime -\beta )\psi (\alpha +\beta ).$$ (1)

where *α* and *β* are the shape parameters of the Beta distribution of NDVI from the original image, *α*′and *β*′ are the parameters of the Beta distribution after spatial averaging, *B* is the beta function, and *ψ*(*x*) is the digamma function:

$$\psi (x)=\frac{d}{dx}ln(\Gamma (x))$$ (2)

where *Γ*(*x*) is the gamma function.

To quantify scaling relationships within the field on the different measurement days we calculated the radially-averaged power spectral density (Y) of each NDVI image [45,46] with Fatiando a Terra v0.5 for Python [47], and interpreted the resulting spectra in terms of its power-law exponent *b* [48,49]:

$$Y=ck^{b}$$ (3)

where *k* is scale (m^−1^) and *c* is a normalization constant.

## Results

### Spatial and temporal patterns of NDVI

**Fig 2:**
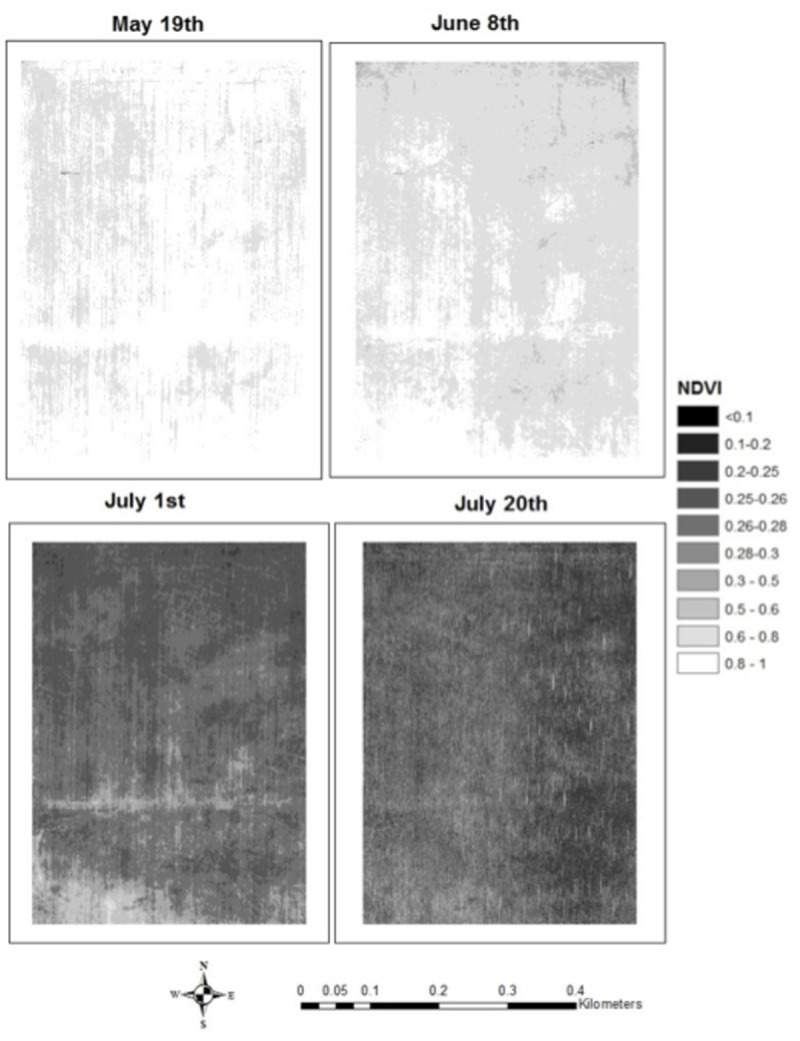
The observed normalized difference vegetation index (NDVI) measured by an unoccupied aerial vehicle (UAV) from a winter wheat field near Sun River, Montana for four measurement dates in 2016.

**Fig 3:**
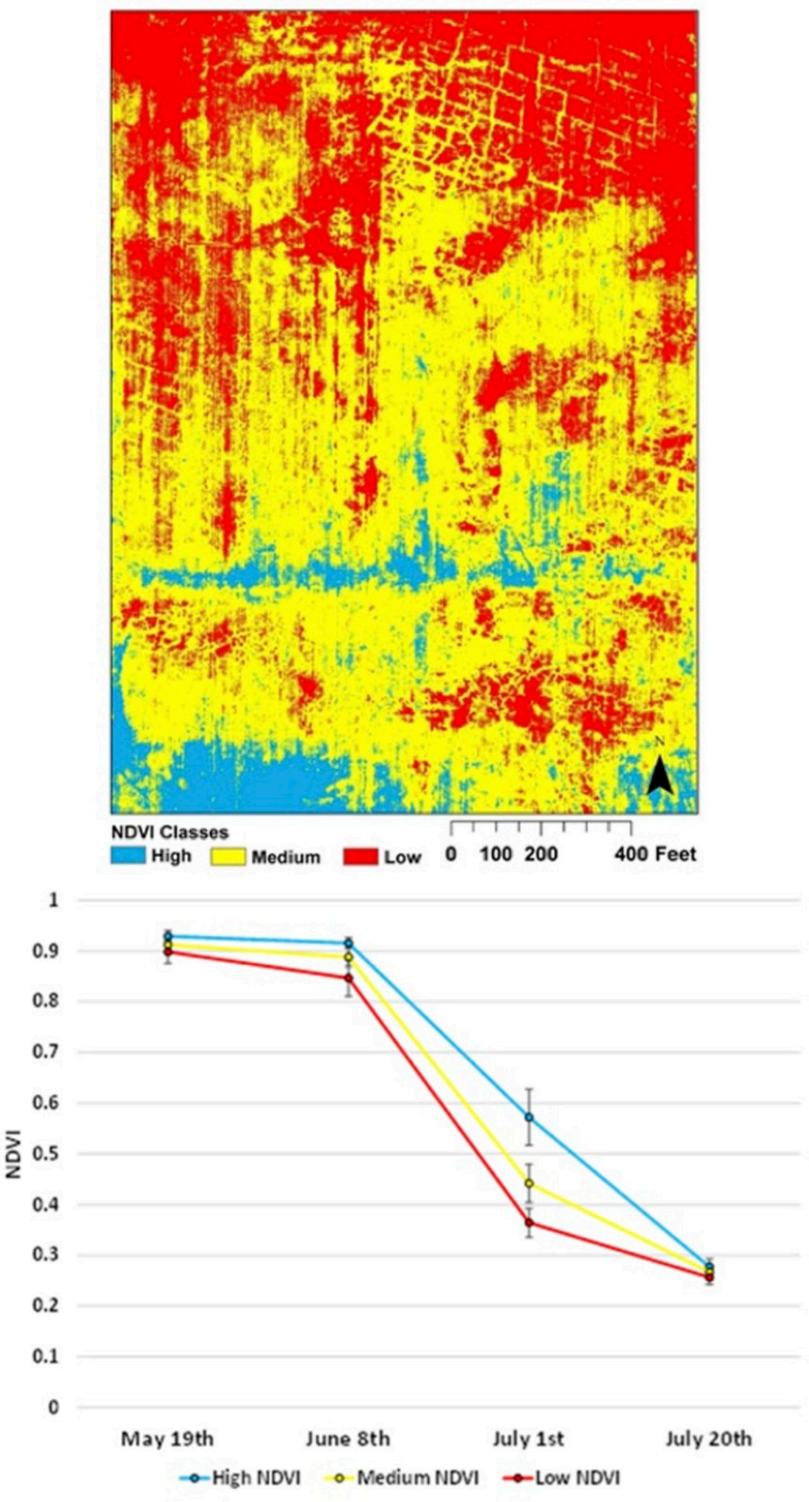
Results of an unsupervised classification of NDVI into relatively high, medium, and low NDVI classes.

NDVI averaged 0.91±0.014 on May 19, 0.88 ± 0.025 on June 8, 0.44 ± 0.063 on July 12, and 0.27 ± 0.011 on July 20 (Fig 2). Unsupervised classification distinguished different parts of the field as having relatively high, medium, or low NDVI trajectories across the growing season (Fig 3). This classification–and the images themselves–reveal NDVI patterns with different characteristic length scales from centimeters to hundreds of meters, with implications for yield, GPC, and within-field management opportunities.

### Relationships between NDVI and wheat yield

**Fig 4:**
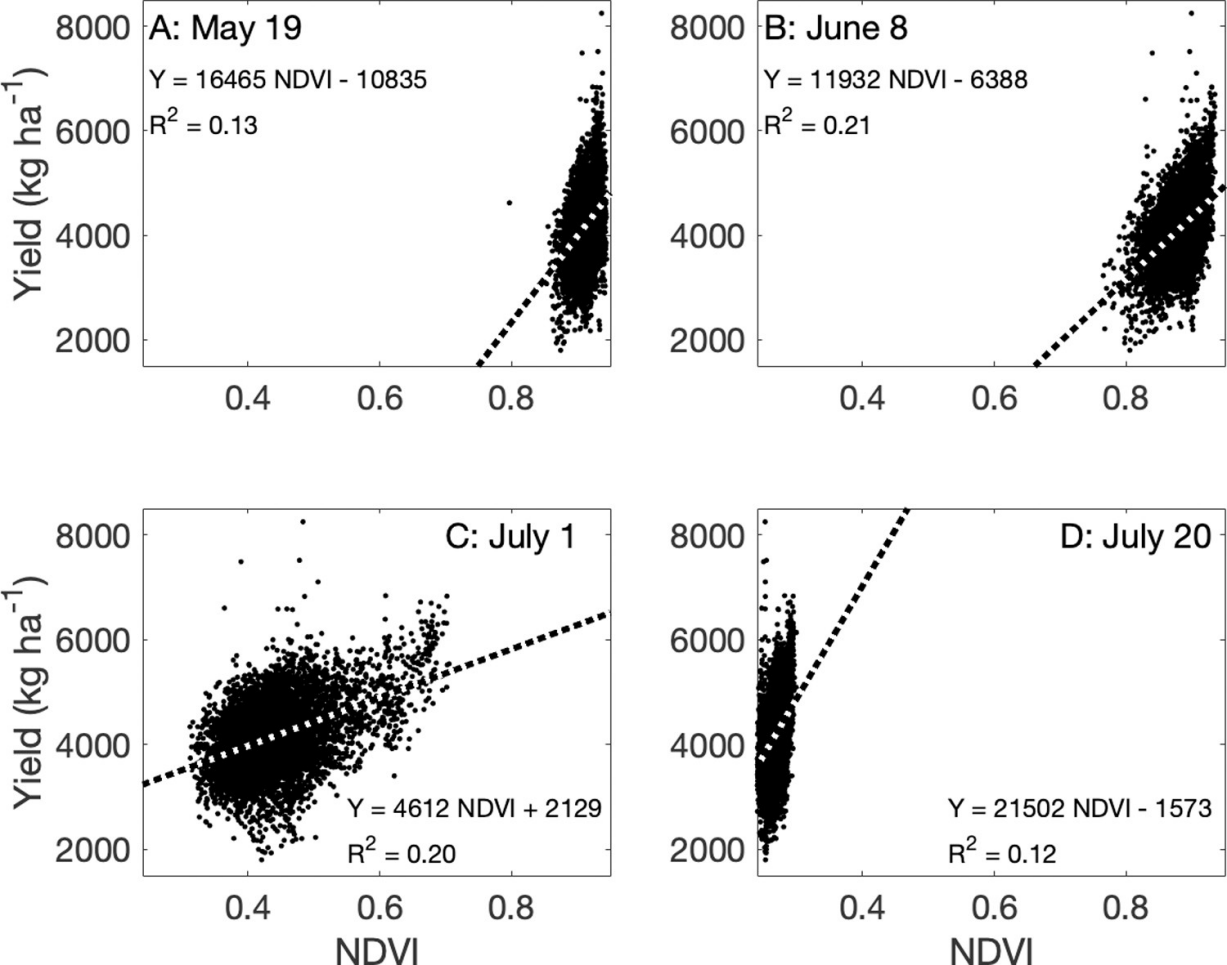
The relationship between the normalized difference vegetation index (NDVI) measured by an unoccupied aerial vehicle on four dates and wheat yield in a winter wheat field near Sun River, MT, USA.

**Fig 5:**
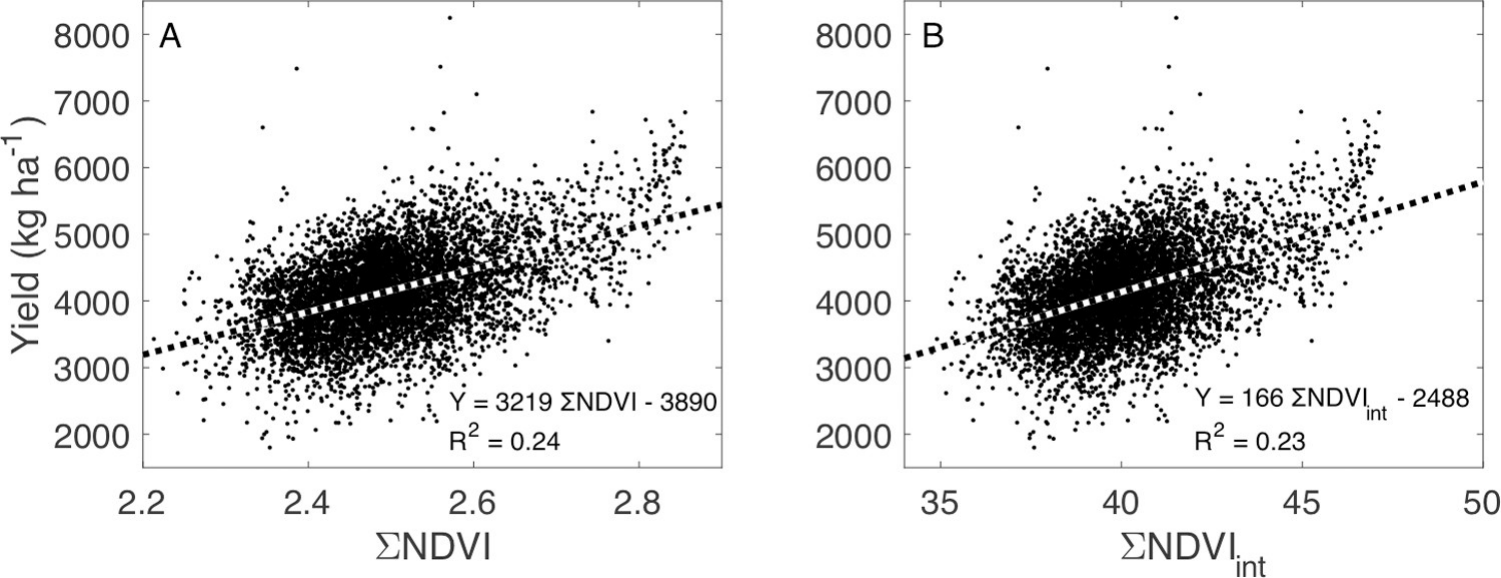
The relationship between winter wheat yield and the sum of unoccupied aerial vehicle measurements of the normalized difference vegetation index (*Σ*NDVI) for four measurement dates in a winter wheat field in Montana, USA (A, see Fig 4). The relationship between yield and the sum of daily NDVI from May 19, 2016, until July 20, 2016 was created with a linear interpolation of NDVI measurements (*Σ*NDVI_int_) across the four measurement dates.

NDVI measurements from each UAV flyover were significantly related to yield (*P* < 0.05, Fig 4), but Landsat NDVI observations only explained 1% of its variability. NDVI measurements from June 8 (NDVI_June8_) and July 12 (NDVI_July12_) explained 20% or more of the variability of wheat yield (Fig 2 top), but NDVI_May19_ and NDVI_July20_ explained less than 14%. Linear model selection using AIC indicated that a model that summed NDVI measurements from all periods (ΣNDVI) explained nearly 25% of the variability in yield (Fig 5A) and represented 59% of the weight–the relative likelihood–across all models tested. Assuming a linear relationship between each NDVI observation and time, creating a NDVI product for every day, and summing the subsequent interpolated values did not improve the model (Fig 5B). The model with the highest *R*^*2*^, Yield = −11520 + 963.2 ×NDVI_July1_ + 3750 ×NDVI_July20_ + 7254 × NDVI_June8_ + 8617 × NDVI_May19_, explained 26% of the observed variability in yield, similar to the linear model as a function of *Σ*NDVI. In other words, a model with four discrete NDVI measurements explained slightly more variability in yield than a measurement that included only their sum but was penalized by the AIC analysis for having more parameters.

### Relationships between NDVI and grain protein content

**Fig 6:**
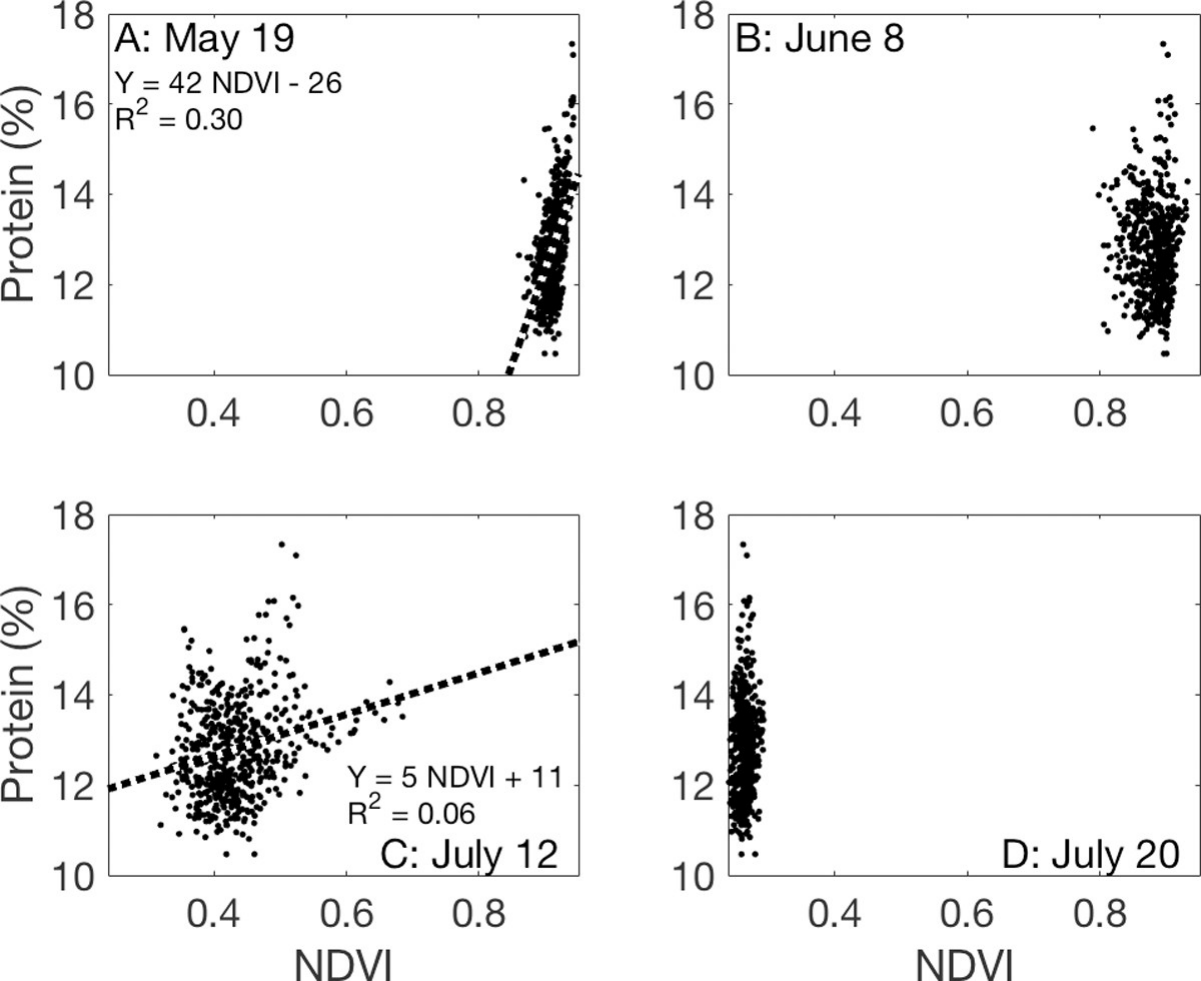
The relationship between the normalized difference vegetation index (NDVI) measured by an unoccupied aerial vehicle and grain protein content in a winter wheat field near Sun River, MT, USA. Relationships that are not significant at the *P* < 0.05 level are not plotted.

NDVI_May19_ explained 30% of the variability in GPC. NDVI_July19_ was also significantly related to GPC (*P* < 0.05) but only explained 6% of its variability (Fig 6). Model selection using AIC chose a model that includes NDVI_May19_, NDVI_July20_, and a negative relationship with NDVI_June8_, but not NDVI_July12_:

$$GPC=-25.20+27.9100\times NDVI_{July20}-19.4100\times NDVI_{June8}+52.36\times NDVI_{May19}.$$

**Fig 7:**
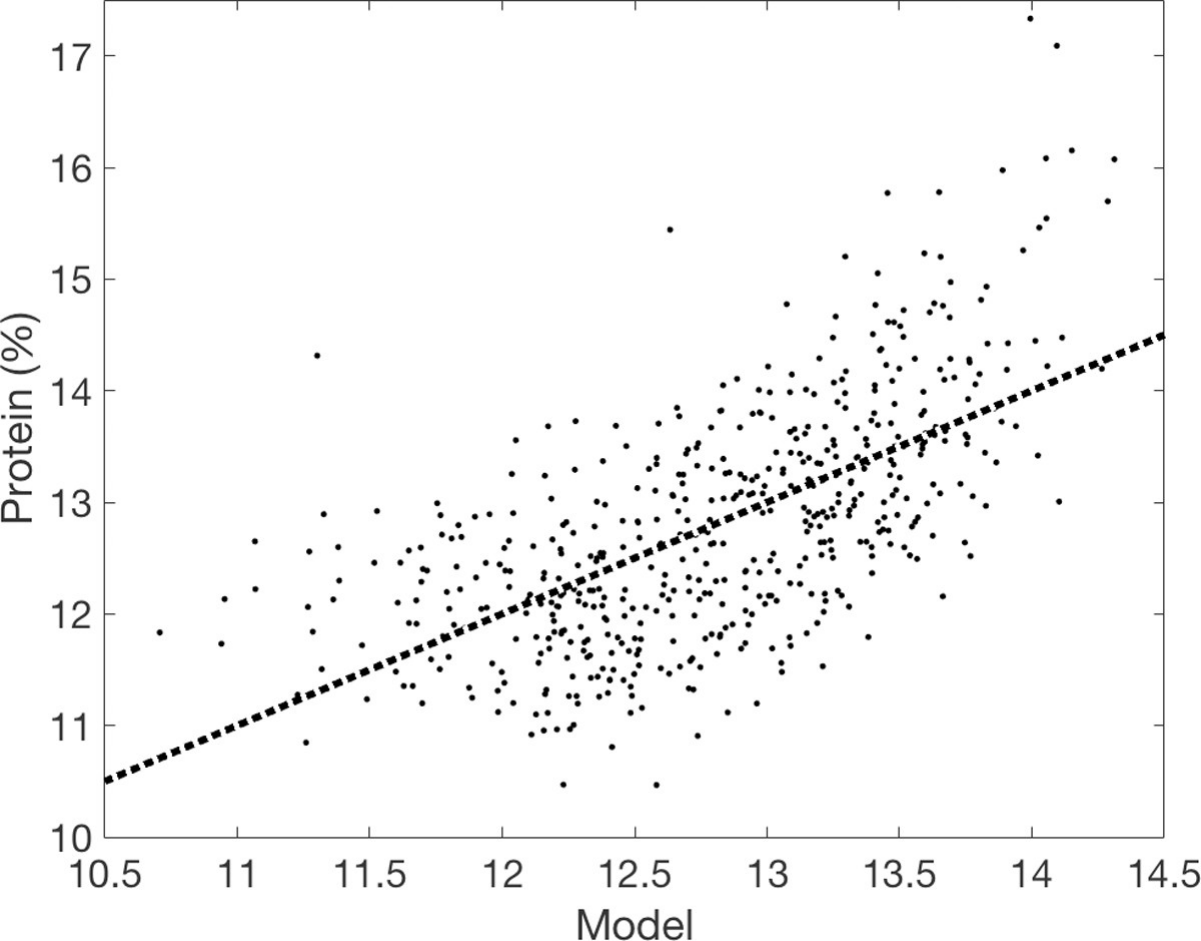
The relationship between protein content (%) and the best-fit linear model of all identified using Akaike’s Information Criterion: Protein = −25.20 + 27.9100× NDVI_July20_ − 19.4100 × NDVI_June8_ + 52.36 × NDVI_May19_. The dashed line represents the 1:1 line.

This model explained 40% of the variability in GPC and represented 59% of the weight across all models tested (Fig 7). The remaining 41% weight was represented by a model that includes NDVI on all dates including a negative term for NDVI_June8_, meaning that the most parsimonious model would be represented by a combination of 59% of the model that included three NDVI dates and 41% of the model that included all four. We also explored Red Edge as an alternative to NDVI, but this explained about 1% less of the variability in GPC than did NDVI and did not improve the yield model.

### Interpreting the NDVI observations as a function of spatial scale

**Fig 8:**
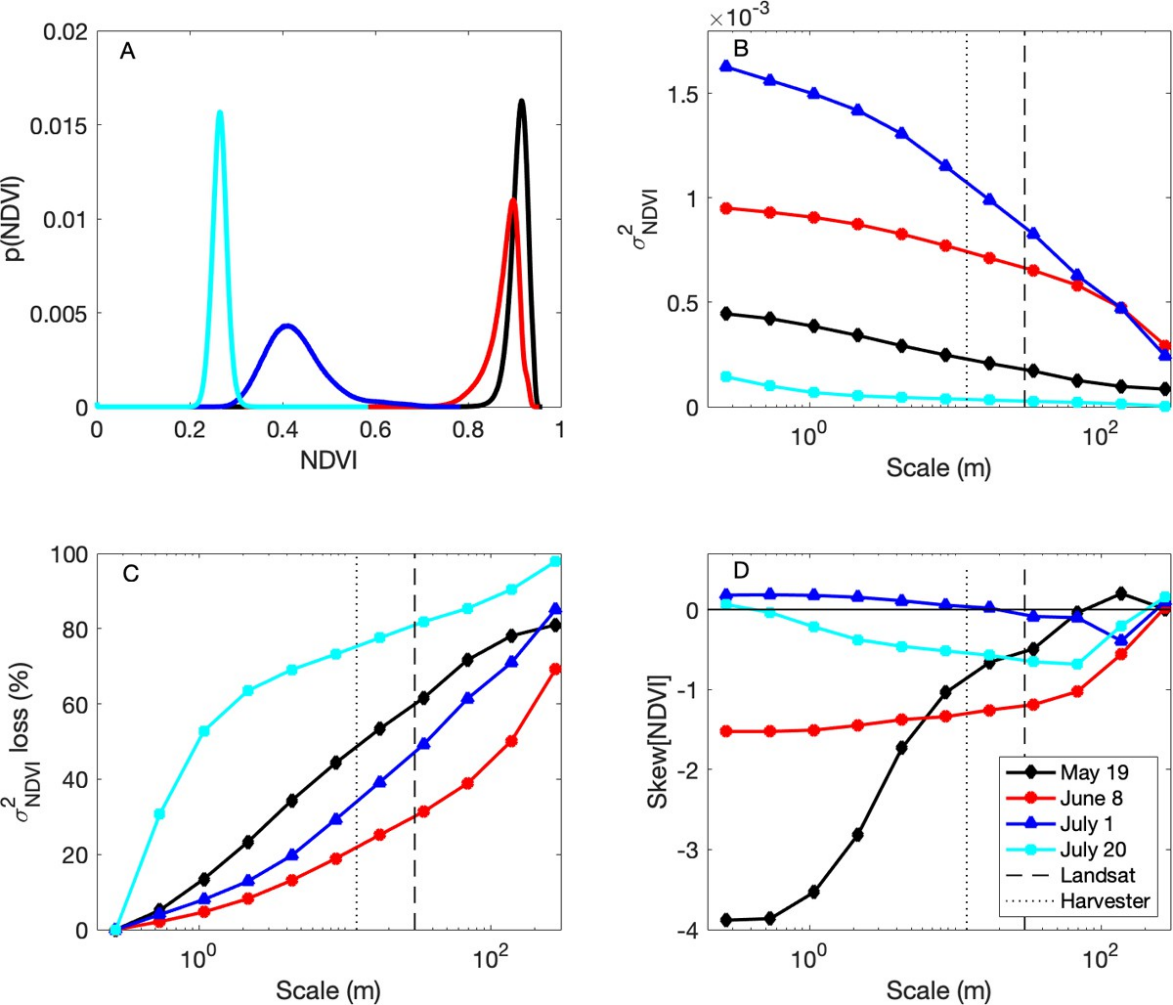
The distribution of the NDVI images (A) and variance (B), loss of variance (C), and skewness (D) of each NDVI image as a function of spatial scale. The 30 m length scale of Landsat (dashed line) and the 12 m length scale of the harvester (dotted line) are indicated for reference.

The rich spatial patterns of NDVI observations (Figs 2 and 3) led us to question how much of the variability in their distributions (Fig 8A) was “averaged out” by Landsat that provided data on 30 m scales and the harvester that provided yield and GPC data on 1 × 12 m scales. Total variance monotonically decreased with larger spatial averaging operators, i.e. as spatial grain size increased for each image (Fig 8B), but with different slopes and degrees of nonlinearity such that the role of averaging may be better envisioned by the loss of variance as a function of scale (Fig 8C). Over 50% (75%) of the total variance of the NDVI_May19_ (NDVI_July20_) image was lost when aggregating to the scale of the harvester and Landsat, but only ⅓ of the total variance of the NDVI_June8_ image was lost at the 30 m Landsat scale. The earlier NDVI measurements (May 19 and June 8) had substantial negative skew (Fig 8D), indicating the presence of areas in the field with far lower NDVI than the mean that are likely candidates for management intervention. This skewness was also “averaged out” at larger spatial scales, especially the NDVI_May19_ image whose skewness changed from −4 to −0.5 upon averaging to the Landsat scale.

**Fig 9:**
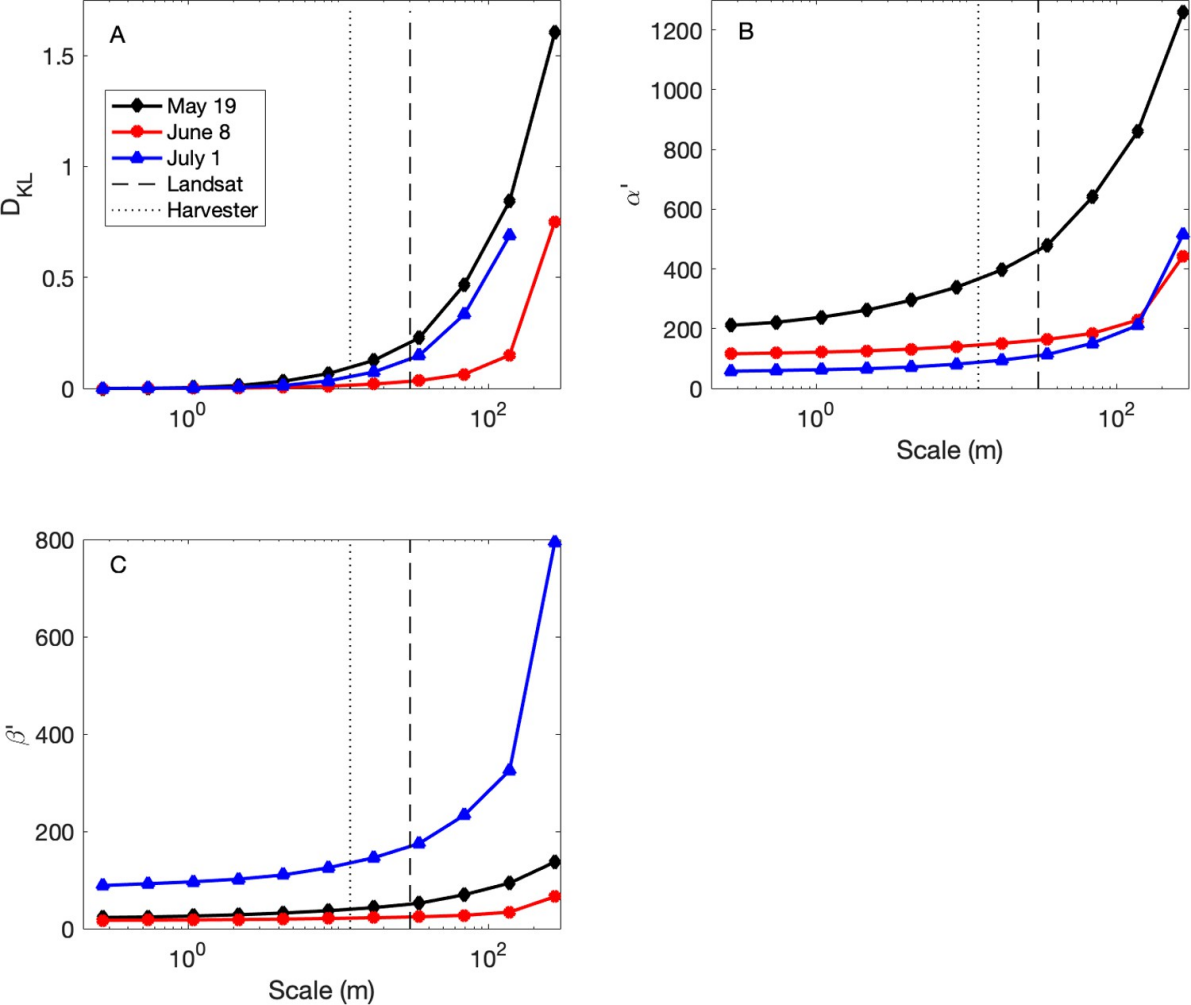
The change in Kullback-Leibler divergence (D_KL_, A), the α parameter of the Beta distribution (α’, B), and the β parameter of the Beta distribution (β’, C) of observed NDVI as a function of spatial scale. The 30 m length scale of Landsat (dashed line) and the 12 m length scale of the harvester (dotted line) are indicated for reference.

The D_KL_ quantifies the change in information content between the original and spatially-averaged images. It increased rapidly at spatial scales larger than 30 m (Fig 9A) but was less than 0.15 (0.25) at the harvester (Landsat) scale for the NDVI_May19_, NDVI_June8_, and NDVI_July1_ images. (The D_KL_ for the NDVI_July20_ image was consistently much larger and is not shown in the figures for clarity.) Changes to the α parameter (i.e. α’) dominated D_KL_ for the May 19 and June 8 images as spatial grain became larger, and changes to the β parameter (i.e. β’) dominated D_KL_ for the July 1 image.

**Fig 10:**
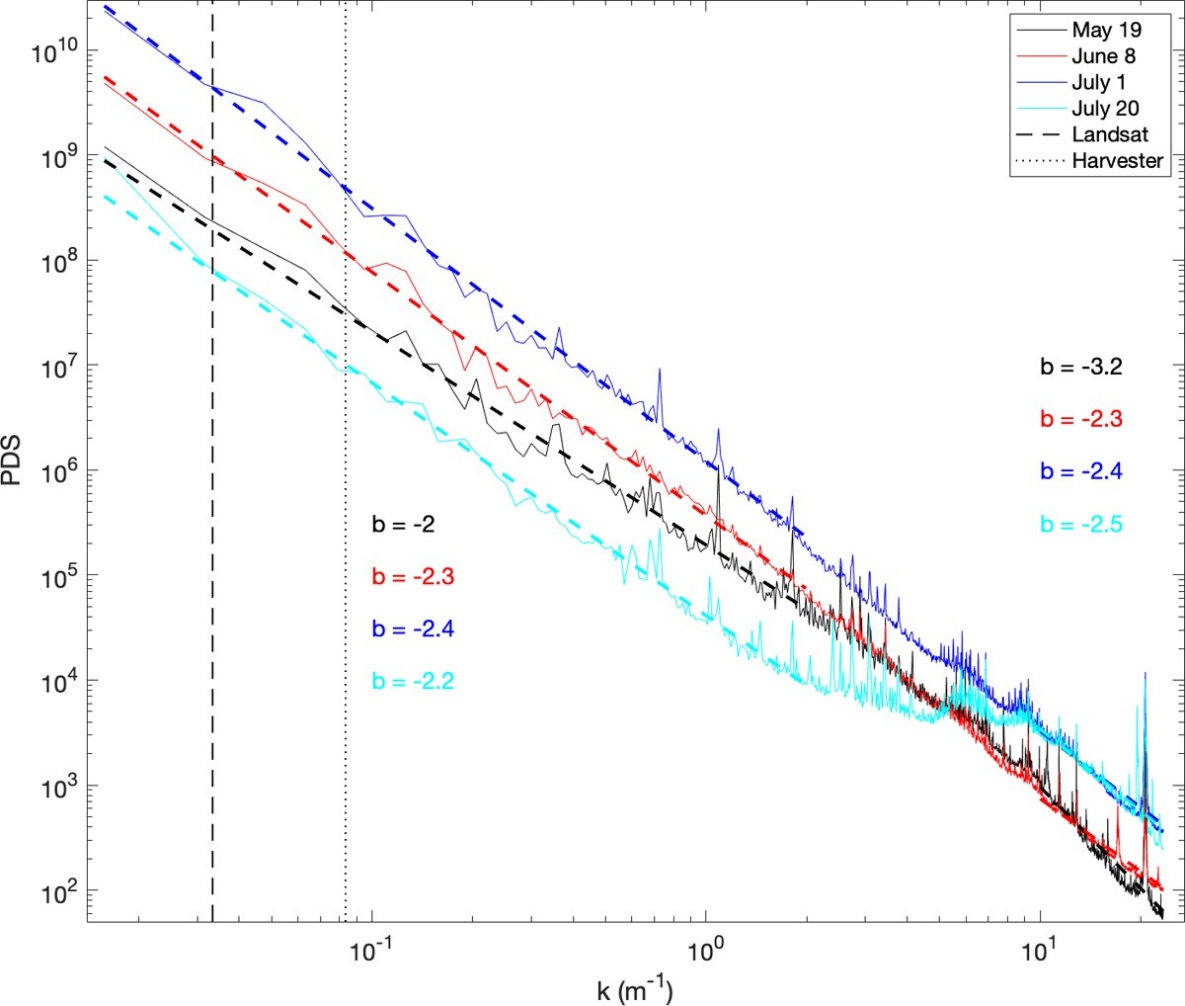
The radially-averaged power density spectra (PDS) of each NDVI image as a function of scale (*k*) with the power law exponent *b* for values less than 2 m^−1^ (left) and greater than 10 m^−1^ (right). The 30 m length scale of Landsat (dashed line) and the 12 m length scale of the harvester (dotted line) are indicated for reference.

The power law exponent (i.e. *b*) of the radially-averaged power-density spectra was constant at *b* = 2.3 (2.4) for the June 8 (July 1) images across all scales (Fig 10) noting that the July 1 image has more total variance than the June 8 image (Fig 8B). There was notable variability in all spectra and a scale break in the May 19 and July 20 images on the order of 6 m^−1^ (i.e. ~17 cm) and *b* decreased faster at spatial frequencies larger than this value, especially in the May 19 image when it decreased from −2 to −3.2 (Fig 10). There was also notable variability in all spectra at 20.6 m^−1^, about 5 cm (Fig 10). Some of the minor peaks at lower spatial frequencies present in the other images were absent in the June 8 image which suffered from less information loss at larger spatial scales than the other images (Fig 8C).

## Discussion

Detailed observations are expected to provide agricultural producers with the knowledge to further develop prescriptive management practices. Because UAV mapping is becoming widespread, it is essential to explore the boundaries of what is practical and necessary to improve agricultural management and sustainable production. We discuss how the interpretation of NDVI at fine spatial scales can provide producers with the correct amount of information–not too much and not too little–to understand within-field variability in important attributes that are related to yield and GPC.

### Spatio-temporal patterns of NDVI

The southwest portion of the study field had consistently higher NDVI throughout the growing season. This part of the field had lower topography that likely benefits from water drainage in characteristically dry north-central Montana (Figs 1 and 3). There was an east-to-west swath of higher NDVI that was identified as an old fence line, where blowing soil likely accumulated in prior decades and improved fertility (Fig 3). Areas of moderately high NDVI were widely distributed throughout the field and were observed along thin linear features, especially in the northeast portion of the field, thought to be associated with the edges of shale cracks from which roots could access deeper soils. Areas of consistently lower NDVI through the growing season were primarily clustered in the northern portion of the field, likely associated with lower water retention and thinner soils at higher topography. Such observations can guide further soil sampling, which is key to further improve yield prediction [50]. Note that these patterns are not readily apparent to the human eye, to which the field appears largely homogeneous (S1 Fig).

From this analysis, it is apparent that NDVI observations provide rich spatial information to producers. The question is how much is necessary. All four UAV flights were necessary to identify key features of the study field; note for example that many of the features identified by the unsupervised classification (Fig 3) were not apparent in the May 19 image (Fig 2A). NDVI measured early in the growing season can predict eventual yield [51] but feature identification relied on all images, as did the best model for yield prediction (Figs 4 and 5). NDVI from the May 19 image alone was able to explain 30% of the variability in GPC (Fig 6A), and additional observations increased predictive power by only about 10% (Fig 7). Management interventions during earlier dates, especially during the wheat heading stage, are candidates for N top dressing, the major within-season management correction that producers can take to enhance GPC [52]. In other words, all the images produced information that can be useful for understanding the idiosyncrasies of an individual field, but earlier information can guide specific management strategies to benefit yield and GPC in a given growing season. One potential approach to maximize information and minimize effort is to make multiple flyovers during initial investigations to understand the properties of individual fields, then reserve flights in future years for early periods of the growing season to identify deficiencies from expected crop growth patterns.

### NDVI as a function of spatial scale

It is readily apparent that the high-resolution information from the UAV flyovers greatly exceeds the yield and GPC information that the harvester can provide, creating a scale mismatch that can be understood by exploring how spatial averaging alters the information content of the NDVI images. At least 22% (June 8) and up to 75% (July 20) of the observed NDVI variance is averaged out at the scale of the harvester, 12 m (Fig 8B and 8C), which makes much of the information content of the UAV NDVI images irrelevant for understanding yield and GPC observations collected at coarser scales. Notably, many of the underperforming areas visible early in the May 19 image by its negative skew (Fig 8D) were averaged out at larger spatial scales. That being said, the practical consequences of high skewness in the case of the study field may be unimportant; less than 0.1% (10,000) of the nearly 12.3 million NDVI_May19_ observations had an NDVI of less than 0.8 on May 19. Instead of dwelling on information loss with spatial averaging, there are many features of NDVI at coarser spatial scales that might be considered promising for a simpler description of its spatial variability.

In addition to the relatively low loss of variance in the June 8 image, the D_KL_ analysis reveals low information loss compared to the other images (Fig 9A). This means that the shape of the Beta distribution, as defined by its parameters (Fig 9B and 9C), was largely maintained upon spatial averaging. In other words, parameters fit from data at coarser spatial scales are a reasonably good approximation for those fit from data at finer scales. It helps that NDVI followed unimodal distributions for all UAV flights.

This opens the possibility for an efficient description of the variability of fine-scale data from coarse-scale data, as also revealed by the scaling analysis (Fig 10) which demonstrates that NDVI from all images follows a power-law scaling relationship of *b* ~ −2 at spatial scales larger than ~ 0.5 m. The June 8 and July 1 images had a common scaling relationship of *b* ~ −2 across all scales. The May 19 image follows an even steeper power-law relationship (*b* ~ −3.2) at spatial scales smaller than ~ 0.1 m suggesting that exponentially less information is present at high frequencies and the dominant modes of variability in the field are at relatively low spatial frequencies, i.e. large spatial scales. These observations demonstrate that small spatial scales make minor contributions to the total variance of the image, but our analysis was constrained by the spatial scale of the harvester, below which we had no basis to infer spatial patterns of yield and GPC. Obtaining harvester information at finer spatial scales would help match the spatial resolution (‘grain’) of the UAV observations and we anticipate that harvester technology will continue to improve to make it cost-effective for producers to invest in instrumentation that can measure grain attributes at finer spatial resolutions.

### Remote sensing at sub-field scales

It is important to note throughout this analysis that we investigated NDVI when multiple indices have proven effective for understanding wheat yield and GPC [53] and it remains unclear which is best [19,54]. Information from green and blue bands tends to be less successful for predicting wheat yield [55] and we found lower descriptive power when using red edge measurements (not shown). Moving beyond NDVI, other vegetation indices like the plant pigment ratio have been successful for predicting GPC [56]. Multispectral data have proven effective for predicting wheat yield [57,58], water status [59], GPC and nitrogen dynamics [60–62], senescence [63], and even detecting diseases [64]. Newer techniques including sun-induced fluorescence show promise in understanding wheat nitrogen dynamics [65,66]. The near-infrared reflectance of vegetation (NIRv) and variations that include reflected radiance show promise for understanding gross primary productivity in heterogeneous ecosystems [67,68]. Combined, our results suggest that not all spectral data are necessary for a concise description of yield and GPC, nor are all spatial data. Going forward, we recommend an experiment that ‘oversamples’ within-field wheat spectral reflectance at hyperspectral, ‘hypertemporal’ [69,70], and hyperspatial resolution to quantify the information that is necessary to predict yield and GPC, as well as the unnecessary information. By quantifying the benefits, but also the costs, of information acquisition, producers can gain a richer understanding of the most cost-effective information to collect to manage wheat yields and GPC and continue feeding a growing populace.

## Supporting information

S1 Fig A photograph of an eddy covariance tower on the study field taken on May 4, 2016, and located at 47.4758 N, 111.7207 W (Image credit: Dr. James Irvine). (DOCX)

S2 Fig Yield and grain protein content (GPC) data from a combine sensor were averaged across 1 × 12 m rectangular buffers to approximate the combine footprint. The dark area in the center of the image is the location of the micrometeorological tower, which was avoided by the combine.
(DOCX)
