# Supplementary material for: The spatial variability of NDVI within a wheat field: Information content and implications for yield and grain protein monitoring
Source: PLoS One. 2022 Mar 22;17(3):e0265243. doi: 10.1371/journal.pone.0265243 (PMC8939815; doi:10.1371/journal.pone.0265243)
Supplement: S1 Fig — (DOCX) [file pone.0265243.s001.docx]

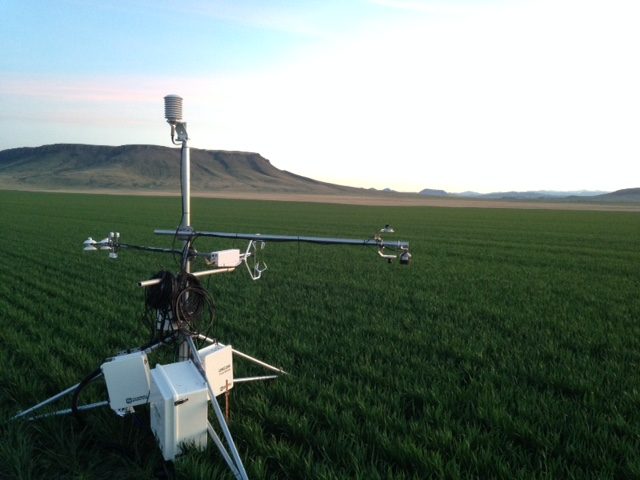


**Fig S1**. A photograph of an eddy covariance tower on the study field taken on May 4, 2016, and located at 47.4758 N, 111.7207 W (Image credit: Dr. James Irvine).
