# Supplementary material for: The spatial variability of NDVI within a wheat field: Information content and implications for yield and grain protein monitoring
Source: PLoS One. 2022 Mar 22;17(3):e0265243. doi: 10.1371/journal.pone.0265243 (PMC8939815; doi:10.1371/journal.pone.0265243)
Supplement: S2 Fig — The dark area in the center of the image is the location of the micrometeorological tower, which was avoided by the combine. (DOCX) [file pone.0265243.s002.docx]

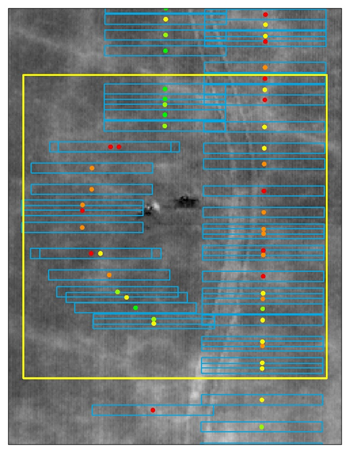
❡

**Fig S2**. Yield and grain protein content (GPC) data from a combine sensor were averaged across 1 $\times$ 12 m rectangular buffers to approximate the combine footprint. The dark area in the center of the image is the location of the micrometeorological tower, which was avoided by the combine.
